# CgNis1’s Impact on Virulence and Stress Response in *Colletotrichum gloeosporioides*

**DOI:** 10.3390/ijms25063505

**Published:** 2024-03-20

**Authors:** Sheng Guo, Qianlong Sun, Sizhen Liu, Fei Wu, Chenggang Li, Xin Zhang, Chao OuYang, Yue Chen, Xinqiu Tan

**Affiliations:** 1 Longping Branch, College of Biology, Hunan University, Changsha 410125, China; 13699547753@163.com (S.G.); qianlongsun153@163.com (Q.S.); l18942563793@163.com (S.L.); wf1041225499@163.com (F.W.); 2 Institute of Plant Protection, Hunan Academy of Agricultural Sciences, Changsha 410125, China; lcgag777@163.com (C.L.); ailuo36@163.com (X.Z.); 17674652278@163.com (C.O.); 3 Yuelushan Laboratory, Changsha 410128, China

**Keywords:** CgNis1, conidiogenesis, growth, pathogenicity

## Abstract

Pepper anthracnose caused by *Colletotrichum gloeosporioides* infection is an important fungal disease and represents a serious threat to pepper yield and quality. At present, the pathogenic molecular mechanism of *C. gloeosporioides* is not very clear. In our study, we characterized the function of *C. gloeosporioides* CgNis1, a homolog of *Magnaporthe oryzae* MoNis1. We found that the ∆*Cgnis1* mutant reduced the growth rate and was defective in conidiation. Although the rate of appressorium formation was unaffected, the germ tube was found to be abnormal. CgNis1 was shown to be involved in the H_2_O_2_ stress response and maintaining cell membrane permeability. The pathogenicity assays performed in this study indicated that the deletion of CgNIS1 is associated with virulence. Our results indicate that CgNis1 is necessary for the growth, development, and pathogenicity of the fungus. This work provides an in-depth analysis of the Nis1 protein, helps to enhance studies on pathogen-related molecular mechanisms, and provides a theoretical basis for the prevention and control of *C. gloeosporioides* in peppers.

## 1. Introduction

Pepper anthracnose is one of the most important fungal diseases in chili production regions around the world. It is widely distributed, highly harmful, and spreads rapidly, seriously threatening the yield and quality of chili peppers [1]. The main pathogenic fungi include *Colletotrichum gloeosporioides* [2], *Colletotrichum truncatum* [3], *Colletotrichum acutatum* [4], *Colletotrichum capsici* [5], and *Colletotrichum brevisporum* [6]. Research has shown that there are significant differences in the dominant populations of pepper anthracnose pathogens in different regions, among which *C. gloeosporioides*, *C. acutatum*, and *C. capsici* are the most widely distributed pathogens of pepper anthracnose fungus [7]. In countries such as Nigeria and India, *C. capsici* is the main pathogenic fungus involved. *C. acutatum* is the main pathogen that causes anthracnose in Korean chili peppers and sweet peppers, while in the main chili-producing areas of central and southern China, the fungus *C. gloeosporioides* is the main dominant population that causes pepper anthracnose disease [8].

Similar to other plant pathogenic fungi, *C. gloeosporioides* also spreads disease through conidia infection. When the conidia of *C. gloeosporioides* come into contact with the host surface, their tips expand to form appressoria [9]. Once the appressoria are fully mature, huge swelling pressure accumulates inside, which generates a penetration peg through compression. The penetration peg can penetrate the host epidermis, invade the interior region of plant cells, and then, differentiate into invading hyphae, which quickly expand to the entire cell and adjacent cells, followed by the appearance of disease spots on the leaves. Under appropriate conditions, these lesions will differentiate and produce a large number of conidia, which will be released from the lesions and begin a new round of infection [10].

Currently, with the continued release of genomic data and the optimization of genetic transformation systems, research on functional genes of *C. gloeosporioides* has become more convenient and efficient. Mitogen-activated protein kinase (MAPK) signaling pathways are now known to be critical for responding to various environmental signals. Research has proven that *C. gloeosporioides* mycelia growth, asexual improvement, pathogenicity, and the maintenance of cell wall integrity require an MAPKKK protein, CgMck1. In one study, the deletion of *CgMKK1* or *CgMPS1,* which were downstream MAPK cascade aspects, confirmed comparable defects to the ∆*Cgmck1* mutant [11]. The cyclic AMP-dependent protein kinase A (cAMP-PKA) pathway is concerned with the growth, improvement, and pathogenesis of phytopathogenic fungi. Inactivation of the gene that encodes the catalytic subunit of cAMP-based protein kinase A *CgPKAC* made *C. gloeosporioides* delay appressorium formation and weakened its pathogenicity [12]. As a cofactor concerned with redox reactions, copper is indispensable in the biological processes of all eukaryotes. Vacuolar copper transporter *CgCTR2* silencing showed a changed *C. gloeosporioides* germination rate and reduced virulence, decreasing H_2_O_2_ stress tolerance [13]. These studies indicate that intracellular proteins play an exceedingly significant role in the growth, development, and pathogenesis of *C. gloeosporioides*, but there are few reports on the biological functions of extracellular proteins.

The innate immune system of plants is divided into two main parts: one for PTI and one for ETI (effector-triggered immunity). PTI is mediated by RLPs (receptor-like proteins), RLKs (receptor-like kinases), and RLCKs (receptor-like cytoplasmic kinase PAMPs), and they mediate regulation. In response to PTI, pathogenic fungi produce effectors. Effector proteins are defined in some articles as small secreted proteins that are cysteine-rich and have a tertiary structure stabilized by disulfide bridges ≤ 300 amino acids in size [14,15,16,17,18,19], but this is inaccurate, as in the case of the effector protein Cmu1 [20]. The absence of detectable immediate homologous proteins outside the genus has also been used to define effectors [21,22,23], and in recent years, it has been shown that some effectors are conserved across a wide range of pathogenic fungi, such as the proteins encoded by the filamentous fungal *NIS1* gene. Libera suggests that any secreted fungal protein may act as an effector protein [2]. Effectors may be toxic secondary metabolites or proteins that kill the host plant, or they may be secreted proteins that protect the fungus, inhibit the host immune response, or manipulate host cell physiology [24]. For example, the effector LysM of *C. gloeosporioides* promotes its virulence against rubber by affecting invasive structures and suppressing chitin-triggered plant immunity, and thus, its virulence against rubber [25]. According to Irieda [26], the wide distribution of homologous effector proteins in a particular pathogen strain and the abundance of homologous effector proteins among different species can be defined as “core” effectors. *NIS1* is a core effector, among which *Colletotrichum orbiculare* (*CoNIS1*), *Colletotrichum higginsianum* (*ChNIS1*), *Colletotrichum tofieldiae* (*CtNIS1*), and *M. oryzae* (*MoNIS1*) have been identified. In a study, *CoNIS1*, *ChNIS1,* and *MoNIS1* were found to better inhibit INF1-induced cell death compared to *CoNIS1* and *ChNIS1* [26], but *MoNIS1* did not induce necrotic lesions in *Nicotiana benthamiana*. The *CoNIS1*-induced programmed death of *N. benthamiana* cells can be inhibited by the *CgDN3* homologue [27]. The deletion of *NIS1* in *C. orbiculare* was found to have no effect on the virulence of its natural host, cucumber, but the transient expression of *MoNIS1* (which does not induce necrosis in *N. benthamiana*) on *N.benthamiana* leaves followed by the inoculation of *C. orbiculare* revealed that *MoNIS1* expression significantly enhanced *C. orbiculare* lesion development. This suggests a role for the conserved effector *NIS1* in the virulence of *C. orbiculare* [26]. In addition, the disruption of *NIS1*-targeting genes in *M. oryzae* resulted in severely reduced virulence in susceptible barley and rice varieties, indicating the importance of the conserved effector *NIS1* for fungal virulence [26]. *CoNIS1* and *MoNIS1* share an effector-mediated strategy both targeting the conserved central PRR-related kinases BAK1 and BIK1 and disrupting plant PTIs through the inhibition of their kinase activity and interference with BIK1–NADPH interactions, thereby establishing infection in the host plant [26]. BIK1–NADPH interactions disrupt plant PTI and thereby establish infection in the host plant [26]. In recent years, *NIS1* has been studied mainly in *Colletotrichum* spp., while other pathogens have only been partially studied in the apple canker pathogen. Two NIS1-like proteins from *Valsa mali*, VmNIS1 and VmNIS2, play different roles in plant recognition and pathogen virulence. VmNIS1 induces programmed cell death in *V. mali*, whereas VmNIS2 inhibits programmed cell death in *V. mali* induced by INF1 [28]. VmNIS1 is a plant immune inducer, and recombinant proteins of VmNIS1 induce a ROS burst and the activation of immune-related genes in *N. benthamiana*, and VmNIS1 promotes plant disease resistance. VmNIS2 inhibits the flg22-triggered ROS-generated plant immune response and is required for the full virulence of *V. mali*, while VmNIS2 is required for the tolerance of *V. mali* to oxidative stress [28]. In addition, similar to CoNIS1 and MoNIS1, VmNIS1 and VmNIS2 also interact with the BAK1 co-receptor in plants. This suggests that NISI from different species may manipulate plant immunity in the same way. However, in-depth studies are needed to establish whether *C. gloeosporioides* has the same effect and how the *NIS1* effectors affect the pathogenicity of *C. gloeosporioides*.

In this study, we identified an NIS1 protein of *C. gloeosporioides*—the CgNis1 protein. Our results indicate that CgNis1 is not only required for vegetative growth and cell membrane permeability but is also involved in conidiogenesis and pathogenicity in *C. gloeosporioides*.

## 2. Results

### 2.1. Identification and Knockout of CgNIS1

**Figure 1:**
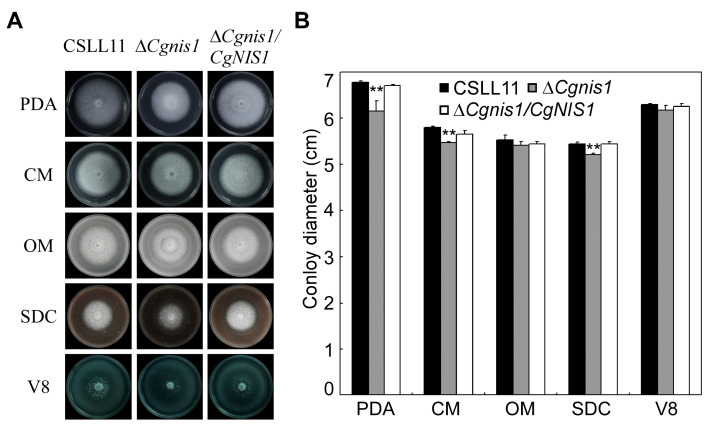
Comparison of mutants and wild-type strains in growth. (**A**) The wild-type, ∆*Cgnis1* mutant, and complementation strains were inoculated on PDA, CM, SDC, OM, and V8 plates and cultured at 28 °C in darkness for 5 days. (**B**) Statistical results of colony diameter of the wild-type, ∆*Cgnis1* mutant, and complementation strains. Standard deviations are represented by error bars. Error bars represent ± SD of three replicates, and asterisks (**) indicate significant difference (*t*-test *p* < 0.01).

**Figure 2:**
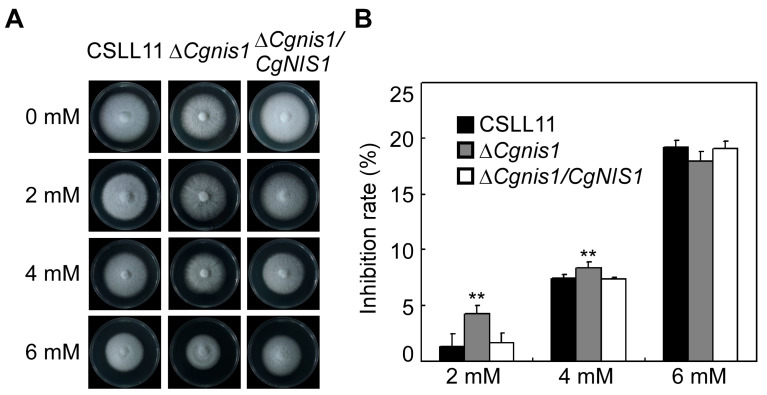
H_2_O_2_ stress assessment of the ∆*Cgnis1* mutants. (**A**) The ∆*Cgnis1* mutants are relatively less sensitive to H_2_O_2_ stress than wild-type CSLL11. Colonies of wild-type CSLL11, ∆*Cgnis1* mutants, and complemented strains were grown on PDA plates with 2, 4, and 6 mM H_2_O_2_ and cultured at 28 °C for 4 days. (**B**) The growth inhibition rate was evaluated relative to the growth rate of each untreated control (inhibition rate = (diameter of untreated strain − diameter of treated strain)/(diameter of untreated strain × 100%)). Similar results were obtained after three repetitions of the procedure. Error bars represent ± SD of three replicates, and asterisks (**) indicate significant difference (*t*-test *p* < 0.01).

**Figure 3:**
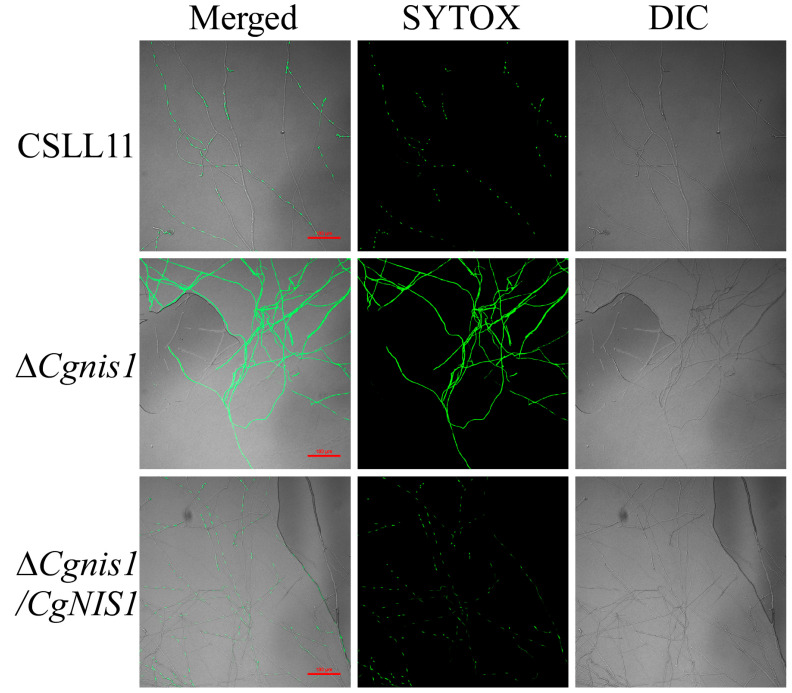
Experimental results of the membrane permeability test on the ∆*Cgnis1* mutant. Approximately 10 µm SYTOX green nucleic acid staining agent was dripped onto the mycelium for 30 min at 37 °C; it was then washed twice with sterile water, and the mycelial fluorescence was observed using a confocal fluorescence microscope and photographed to record the results.

**Figure 4:**
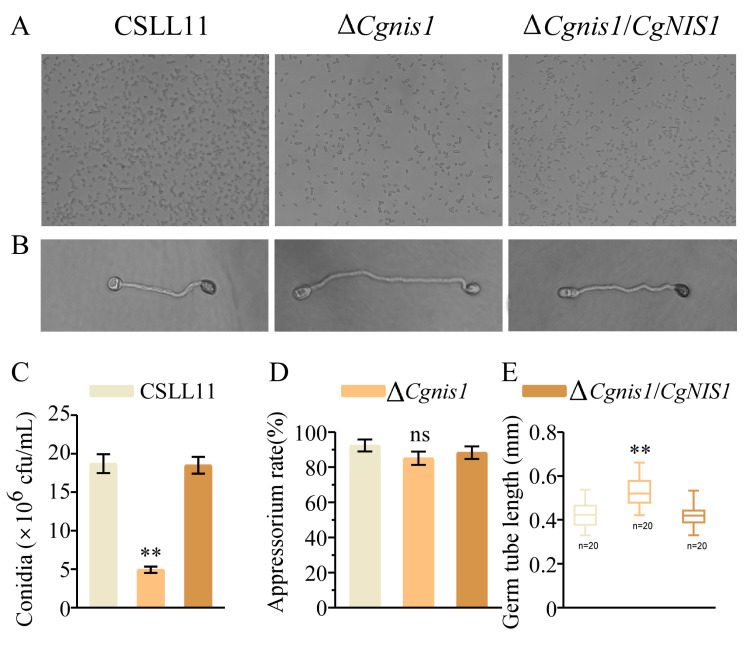
*CgNis1* is required for conidiation. (**A**) Strains were incubated in CM liquid for 3 days to collect conidia for observation under a light microscope. Microscopic images taken at ×20 magnification. (**B**) Morphological observations of appressorium formation. Microscopic images taken at ×40 magnification. (**C**) Statistics on conidial formation quantity (n = 10). (**D**) Statistics on appressorium formation rate. We counted 100 germinating conidia. (**E**) Statistics on germ tube length. Similar results were obtained after three repetitions of the procedure. Error bars represent ± SD of three replicates, and asterisks (**) indicate significant difference (*t*-test, *p* < 0.01).

**Figure 5:**
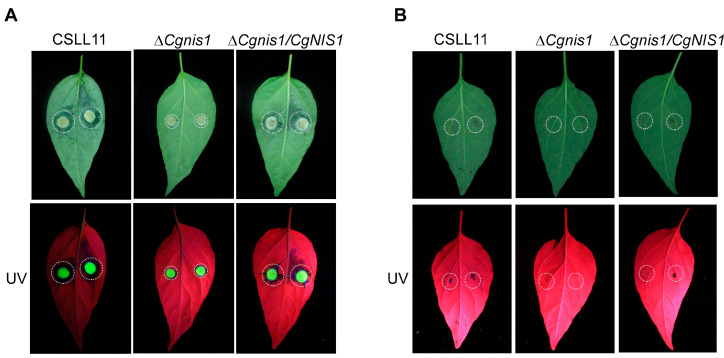
Pathogenicity of the ∆*Cgnis1* mutant on pepper leaves. (**A**) Inoculated round mycelium block of the wild-type CSLL11, ∆*Cgnis1* mutant, and complementation strains on pepper leaves pictured after 3 days. (**B**) Inoculated conidia of the wild-type CSLL11, ∆*Cgnis1* mutant, and complementation strains on the pepper leaves observed after 5 days. UV, ultraviolet light.

**Figure 6:**
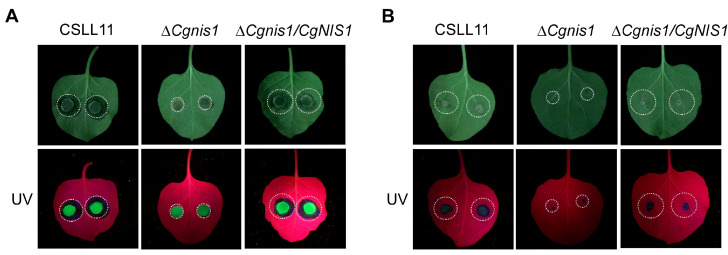
Pathogenicity of the ∆*Cgnis1* mutant on tobacco leaves. (**A**) Inoculated round mycelium block of the wild-type CSLL11, ∆*Cgnis1* mutant, and complementation strains on the tobacco leaves observed after 3 days. (**B**) Inoculated conidia of the wild-type CSLL11, ∆*Cgnis1* mutant, and complementation strains on the tobacco leaves after 5 days. UV, ultraviolet light.

Previous studies have shown that the absence of the *MoNIS1* gene (MGG_02347) significantly reduces the virulence of rice blast fungus on rice and barley [26]. The examination of the *C. gloeosporioides* CSLL11 genome database revealed that a protein encoded by the gene Cghn04645 showed 31% identity and 49% similarity to the *M. oryzae* MoNis1. Consequently, the protein was named CgNis1. We replaced the *CgNIS1*-coding region with the hygromycin-resistance cassette (*HPH*) to generate a *CgNIS1*-deletion mutant. The screening of a hygromycin-resistant colony confirmed a putative knockout mutant (∆*Cgnis1*) through Southern blot analysis (Figure S1B). Upon introducing the *CgNIS1* gene back into the ∆*Cgnis1* mutant, the transformant was confirmed to have normal growth, conidiation, and infection, with the complemented strain ∆*Cgnis1/CgNIS1* (Figure 1, Figure 2, Figure 3, Figure 4, Figure 5 and Figure 6).

### 2.2. CgNIS1 Deletion Affects Vegetative Growth

Initially, mutant growth was assessed on potato dextrose agar (PDA), complete medium (CM), straw decoction and corn agar (SDC), oatmeal medium (OM), and V8 juice agar (V8) plates. On PDA, CM, and SDC media, the ∆*Cgnis1* mutant showed a slightly smaller colony diameter compared to the wild-type strain and the complemented strain ∆*Cgnis1/CgNIS1* (Figure 1A). We counted the colony diameters on different media and analyzed the statistics using a t-test, and the analysis supported this result (Figure 1B), which indicates that CgNis1 is likely to be involved in hyphal growth.

### 2.3. CgNis1 Is Important in H_2_O_2_ Responses

To investigate whether the ∆*Cgnis1* mutant suffers a defect during H_2_O_2_ treatment, we exposed the ∆*Cgnis1* mutant strains to 2, 4, and 6 mM H_2_O_2_. The growth of ∆*Cgnis1* was significantly inhibited at 2 and 4 mM H_2_O_2_ (Figure 2A,B). At 6 mM H_2_O_2_, no significant difference was observed between the ∆*Cgnis1* mutant and the wild-type CSLL11 (Figure 2A,B). These results suggest that CgNis1 may be involved in the oxidative stress response.

### 2.4. CgNIS1 Deletion Affects Cell Membrane Permeability

A SYTOX green membrane permeabilization assay [29] was performed to determine whether *CgNIS1* deletion affects the membrane integrity of *C. gloeosporioides*. The entire hyphae of the ∆*Cgnis1* mutant were observed to display bright green fluorescence after staining with SYTOX for 30 min, while the wild-type CSLL11 and complemented strain ∆*Cgnis1/CgNIS1* showed weak green fluorescence (Figure 3), which indicates that the cell membrane of the ∆*Cgnis1* mutant is damaged, and a large amount of dye enters the cell.

### 2.5. CgNis1 Is Involved in Conidiogenesis but Not Appressorium Formation

Conidia are crucial to the process of *C. gloeosporioides* infection; hence, we measured the ∆*Cgnis1* mutants’ conidia yield. We discovered that on CM liquid medium, the yield of conidia was greatly decreased (Figure 4A). Compared to the wild-type CSLL11 and complemented strain, the conidium yield of ∆*Cgnis1/CgNIS1* was less than one-third (Figure 4C). We further investigated the formation of the appressorium in the ∆*Cgnis1* mutant. We found that the ∆*Cgnis1* mutant had longer germ tubes than the wild type and that it was delayed by 2 h in appressorium formation (Figure 4B,E), but the appressorium formation rate in the ∆*Cgnis1* mutant was not significantly different from that in wild-type CSLL11 and the complemented strain ∆*Cgnis1/CgNIS1* (Figure 4B,D). These results show that CgNis1 is essential to conidiogenesis and alters normal appressorium growth.

### 2.6. CgNis1 Is Required for Full Virulence

To determine whether CgNis1 is involved in pathogenicity, mycelial blocks and conidial suspensions of the ∆*Cgnis1* mutant, wild-type, and complemented strains were placed on pepper and tobacco leaves. The results showed that there was a large water-soaking lesion on the leaves of the peppers 3 days after inoculation with the mycelium of wild-type and complemented strains, while the lesion area in the ∆*Cgnis1* mutant strain was significantly reduced (Figure 5A). On the fifth day after spore inoculation, gray-white lesions could be observed on the leaves infected with wild-type and complemented strains, while the lesion area on the mutant was significantly lower than that on the wild type (Figure 5B). The pathogenicity of tobacco yielded similar results (Figure 6A,B). Altogether, these results suggest that CgNis1 is involved in *C. gloeosporioides* pathogenicity.

### 2.7. CgNis1 Induces Cell Necrosis

**Figure 7:**
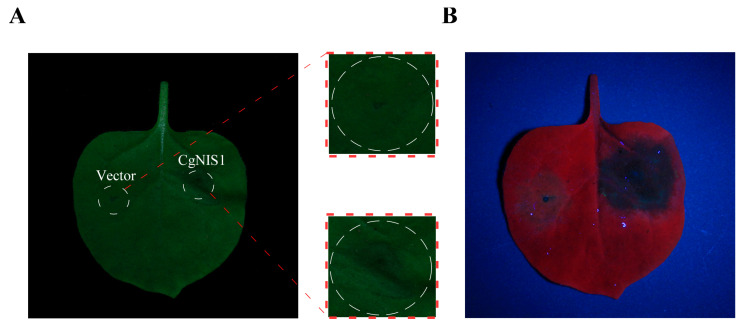
Nis1 induces plant cell death. (**A**,**B**) This picture was taken 42 h after the inoculation of *N. benthamiana*. The left side of the leaves was inoculated with *Agrobacterium tumefaciens* containing the empty vector as the control, and the right side was inoculated with *A. tumefaciens* containing the fusion expression vector pBin::*CgNIS1*::eGFP as the treatment group. (**A**) The picture under white light. (**B**) The picture under ultraviolet light.

In order to verify whether the secreted protein encoded by the *NIS1* gene can induce cell death, we performed a tobacco cell necrosis assay. After inoculation, the leaves of tobacco inoculated with the fusion expression protein showed watery necrotic lesions, while the control group (empty vector) showed only small signs of necrosis (Figure 7A); similar results were observed under UV light irradiation (Figure 7B). These results show that CgNis1 can induce cell death.

## 3. Discussion

NISI proteins are highly conserved in *Colletotrichum* spp.; in addition, *Trichoderma arundinaceum*, *Trichoderma asperellum*, and *Fusarium albosuccineum* also contain NIS1 protein (Figure S2). NIS1 was first studied in *C. orbiculare* (*CoNIS1*) [27] and proved to be the effector. Effector proteins have been demonstrated to control fungal development and growth. One example of this is the ∆*PeLysM3* mutant in *Penicillium expansum*, which shows a somewhat slower rate of radial growth [30]. Followed by the mining of *C. higginsianum* (*ChNIS1*) through NCBI sequence comparison, *C. tofieldiae* (*CtNIS1*), and *M. oryzae* (*MoNIS1*) [26] effectors, functional studies were conducted accordingly. CgNis1 was mined based on the reported *C. orbiculare* NIS1 (AB669517.1) against *C. gloeosporioides* genome comparison. The amino acid sequences of CgNis1 and CoNis1 are highly homologous and conserved, and CgNis1 has a signaling peptide and transmembrane structural domain. Therefore, we hypothesized that CgNis1 is an effector. Our findings indicate that the NIS1 gene regulates nutrient uptake and utilization capacity, and NIS1 deletion leads to a slowdown in the mycelial growth rate of *C. gloeosporioides*. Furthermore, we discovered that ∆*Cgnis1* mutant strains impact cell membrane integrity in addition to displaying hypersensitivity to H_2_O_2_. CoNis1 and MoNis1 inhibit fungal PAMP chitin-triggered ROS production in *N. benthamiana* [26], and *CgNIS1* deletion in our study resulted in enhanced sensitivity to H_2_O_2_; therefore, we hypothesized that the reduced virulence of the deletion mutant might be a result of its high sensitivity to plant defense-related reactive oxygen species (ROS). 

As with most fungal pathogens, the formation of conidia and appressoria plays a significant role in the infection stage. Similar to the results of *CgLysM* in *C. gloeosporioides* [25], the ∆*Cgnis1* mutant showed a decrease in spore production compared with the wild type, suggesting that CgNis1 is involved in conidial formation. The appressorium formation time of the ∆*Cgnis1* mutant was delayed by 2 h compared with the wild type, but the formation rate of appressorium was not affected. These results indicate that CgNis1 positively regulates conidial formation but does not affect the formation of appressorium. Whether it affects the formation of melanin and the turgor size of appressoria still needs to be further explored in the future.

It has been demonstrated in earlier research that secreted proteins affect the pathogenicity of pathogenic fungi during the infectious phase [26,31]. For example, the virulence of the apple ulcer pathogen (*Valsa mali*) is influenced by two Nis1-like proteins called VmNis1 and VmNis2 [28]. The secreted protein encoded by the *NIS1* gene inhibits plant immunity, usually by interacting with the PRR-associated kinases Bak1 and Bik1 to inhibit their kinase activity and subsequent interaction with Bik1-NADPH oxidase [26,32,33]. It has been shown that *NIS1* is preferentially expressed both during appressorium formation and during biotrophic mycelium invasion [27]. In other host–fungus interactions, the presence of diffusible pathogenic effectors to suppress host defense responses has been previously reported [34]. In our study, the sensitivity of the ∆*Cgnis1* mutant to H_2_O_2_ stress may predict its role in virulence, and the fact that the ∆*Cgnis1* mutant has reduced pathogenicity on healthy pepper leaves suggests that there are other potential mechanisms affecting the infection process. CgNIS1 shows a similar function to VmNIS2, both of which are required for complete virulence and tolerance to oxidative stress in *C. gloeosporioides* and *Valsa mal* pathogenic fungi, respectively. CoNIS1 and MoNIS1 manipulate plant immunity by targeting the PTI signaling-associated receptor BAK1, whereas VmNIS1 and VmNIS2 similarly interact with the BAK1 co-receptor in plants [26,28]. This implies that NISIs from different species may manipulate plant immunity in the same way, and we therefore speculate that the effects of CgNIS1 on plants may also be related to the BAK1 signaling pathway. It is unclear how the Nis1 protein interacts with the host during infection. Further in-depth examination of the infection experiment and time course of infection is required. It will be interesting to ascertain whether the infection structure’s cell-dwelling development time has somewhat changed.

Thus far, many pathogens have been identified through the *A. tumefaciens*-mediated transient expression system in *N. thamiana* [35]. A previous study revealed that the transient expression of *C. orbiculare NIS1* caused necrotic formation in *N. thamiana* [27]. In a wide range of fungi in both Ascomycota and Basidiomycota, *NIS1* has a conserved function that targets the key kinases BAK1/SERK3 and BIK1, which are indispensable for plant pattern-triggered immunity (PTI) [26,32,36,37,38,39,40]. This is consistent with our study; therefore, we speculate that the *NIS1* gene may act as an effector protein to interfere with the immune response of plants. Other fungus effector proteins play various roles in some pathogenicity-related physiological processes. However, INF1-induced allergic response (HR) cell death was reduced by the *NIS1* homologues of *C. orbiculare*, *C. higginsianum*, and *M. oryzae* [26]. These findings imply that Nis1 functions differently in various strains. In addition to the widely utilized *N. benthamiana*, it will be crucial to build additional transient expression systems in matching host plants for a deeper understanding of the effector function of plant diseases.

## 4. Materials and Methods

### 4.1. Strains and Culture Conditions

The wild type used in this study is the *C. gloeosporioides* strain CSLL1. CSLL-11 was isolated from anthracnose-affected plants of chili peppers in Changsha, Hunan Province. CSLL11 grows well at temperatures of 25–28 °C and colonies appear as white aerial mycelium. CSLL11 lesions on chili fruits appear as black round depressions or necrosis. All strains were incubated on PDA medium plates in the dark at 28 °C. Liquid CM medium was used to harvest fungal mycelia at 28 °C and 200 rpm, and was used to extract genomic DNA and RNA. The protoplast preparation and transformation processes followed those of Sweigard et al. (1992) [41]. Transformants were selected on media with 400 µg/mL hygromycin B (Roche, Rotkreuz, Switzerland) or 400 µg/mL G418 (Invitrogen, Carlsbad, CA, USA) in TB3 medium.

### 4.2. CgNIS1 Gene Disruption and ∆Cgnis1 Mutant Complementation

For the generation of the *CgNIS1* gene replacement constructs, we used the ligation PCR approach [42]. Primer pairs *NIS1*-UP-F^+^/*NIS1*-UP-R^+^ and *NIS1*-DOWN-F^+^/*NIS1*-DOWN-R^+^ (Table S1) were designed to amplify the sequences of the *CgNIS1* gene approximately 1 kb upstream and downstream by means of PCR. Digestion resulted in PCR products which were amplified by *NIS1*-UP-F^+^/*NIS1*-UP-R^+^ and *NIS1*-DOWN-F^+^/*NIS1*-DOWN-R^+^ primer pairs and separately with XhoI/SalI and SpeI/NotI and purified afterward for ligation to the vector pCX62. We transformed the *CgNIS1* gene replacement constructs into CSLL11 protoplasts. The method used to initially identify putative ∆*Cgnis1* mutants was PCR, and Southern blot analysis was used to further confirm the changes. To generate the complementation strain, the full length of the *CgNIS1* gene was amplified, which contained the native promoter, and we used the digestion–ligation method to clone into the geneticin-resistant vector pGTN and transformed it into the ∆*Cgnis1* mutant. PCR amplification verification was used to obtain *CgNIS1* gene complement transformants.

### 4.3. Southern Blotting

The Southern blot protocol was utilized under the standard protocol [43]. The probes used for Southern blotting were the target gene probe and the *HPH* probe that were amplified with the primer pairs *NIS1*-F/*NIS1*-R (for *CgNIS1*) (Table S1) and *HPH*-F/*HPH*-R (for *HPH*), respectively. The DIG High Prime DNA Labeling and Detection Starter Kit (Roche Applied Science, Penzberg, Germany) was used for probe labeling, hybridization, and detection.

### 4.4. Vegetative Growth, Stress Response, and Cell Membrane Permeability

The vegetative growth of ∆*Cgnis1*, complement strains, and CSLL11 was measured on PDA, CM, OM, SDC, and V8 media for 5 days. Five-day-old mycelia plugs of the same size were cultured in CM liquid for 3 days, with 200 rpm shaking at 28 °C. All growth assays were repeated three times, with three replicates on each occasion.

To determine the effects on the fungal growth of H_2_O_2_, we prepared H_2_O_2_ plates with different concentrations by adding different amounts of H_2_O_2_ solution to the melted PDA medium. Mycelia plugs of the size, 5 mm × 5 mm, were transferred onto the above-mentioned plates and cultured in the dark at 28 °C. Colony size measurements and photographs were taken after 4 days of incubation [40]. The inhibition rate was determined by the percentage decrease in colony diameter [44]. The experiment was repeated three times with three replicates on each occasion. 

Fungal hyphae were added to 20 µL of 10 µm of SYTOX green nucleic acid stain (Thermo Fisher Scientific, Waltham, MA, USA), reacted at 37 °C in the dark for 30 min, and washed twice with sterile water, and then, the hyphae were observed using a confocal fluorescence microscope, and photographs were taken to record the results.

### 4.5. Conidiation and Appressorium Formation

For conidiation, equal volumes of mycelium were incubated for three days at 28 °C and 200 rpm in a 100 mL CM liquid medium. The mixture was then filtered through a 3-layer filter, and 20 µL of the conidial suspension was counted under a microscope using a hemocytometer. To measure the formation of appressoria and conidial germination on a hydrophobic surface, 20 µL conidial suspension (5 × 10^4^ cfu/mL) was dropped onto the surface and placed in a moistened box at 25 °C. The rate of appressorium formation was determined using a microscope at 12 hours post inoculation (hpi), with more than 200 appressoria counted for each strain. Photographs were taken at 12 hpi.

### 4.6. Pathogenicity Analysis of the Gene Knockout Mutants

The tobacco and pepper seeds were sown in advance and cultivated to the 4–6-leaf stage in a greenhouse. Leaves of the same age were selected as test materials. A round mycelium block of the same size and 20 µL spore (5 × 10^4^ cfu/mL) of CSLL11, ∆*Cgnis1,* and complement strains were placed on the leaves and incubated for 3 days (mycelium) and 5 days (conidia) at 28 °C in a dark incubator, and then, photographs were taken to record the results.

### 4.7. CgNis1 Induces Cell Necrosis

The cDNA sequence of the *CgNIS1* gene was amplified via PCR with the primer pairs *NIS1-GFP-F*/*NIS1-GFP-R* and digested with *Kpn*I/*Sma*I, and then, purified and ligated to the vector pBin::eGFP. The connecting vector plasmid was transferred into *A. tumefaciens* GV3101 via electric shock transformation. The *A. tumefaciens*-inoculating buffer containing pBin::Cg*NIS1*::eGFP (treatment group) and the pBin::eGFP (control group) plasmid were injected into the 3-week-old tobacco leaves. After being placed under white light for 1 h, the cultures were switched to darkness for 42 h, after which photographs were taken, and we made a record of the size of the spot.

### 4.8. Statistical Analyses

All biotechnological experiments were repeated thrice using three independent biological replicates. The data were expressed as means ± SD. Shapiro–Wilk was used to analyze the normality among the groups, followed by t-test for evaluation. All statistical analyses were performed using IBM SPSS 21. *p* < 0.05 was considered to indicate statistical significance.

## 5. Conclusions

*C. gloeosporioides* can infest a variety of hosts, including chili peppers [45], bananas [46], strawberries [47], and rubber trees [48]. In this study, we preliminarily investigated the effects of CgNIS1 on the growth, development, and virulence of *C. gloeosporioides*. We created an ∆*Cgnis1* mutant and identified its biological function. The study findings showed that the deletion of *CgNIS1* led to a reduction in hyphal growth rate, conidia yield, and virulence, increased susceptibility to H_2_O_2_, affected appressorium germ tube length, and disrupted cell membrane integration. In conclusion, this research aided in revealing the pleiotropic role of *NIS1* in the control of growth and development as well as pathogen virulence. NIS1 is a core effector that targets PAMP recognition and signaling mechanisms, and these mechanisms may be generally conserved in higher plants [26]. Therefore, we used CgNIS1 as an entry point to lay the foundation for the recognition mechanism of *C. gloeosporioides* and the host plant, as well as to provide targets for resistance breeding and the development of novel drugs for the prevention and control of *C. gloeosporioides*.

## Data Availability

Data are contained within the article and Supplementary Materials.

## Supplementary Materials

The supporting information can be downloaded at: https://www.mdpi.com/article/10.3390/ijms25063505/s1.
