# Supplementary material for: CgNis1’s Impact on Virulence and Stress Response in Colletotrichum gloeosporioides
Source: Int J Mol Sci. 2024 Mar 20;25(6):3505. doi: 10.3390/ijms25063505 (PMC10971101; doi:10.3390/ijms25063505)
Supplement: Supplementary file 1 [file ijms-25-03505-s001.zip › ijms-2906187-supplementary.pdf]

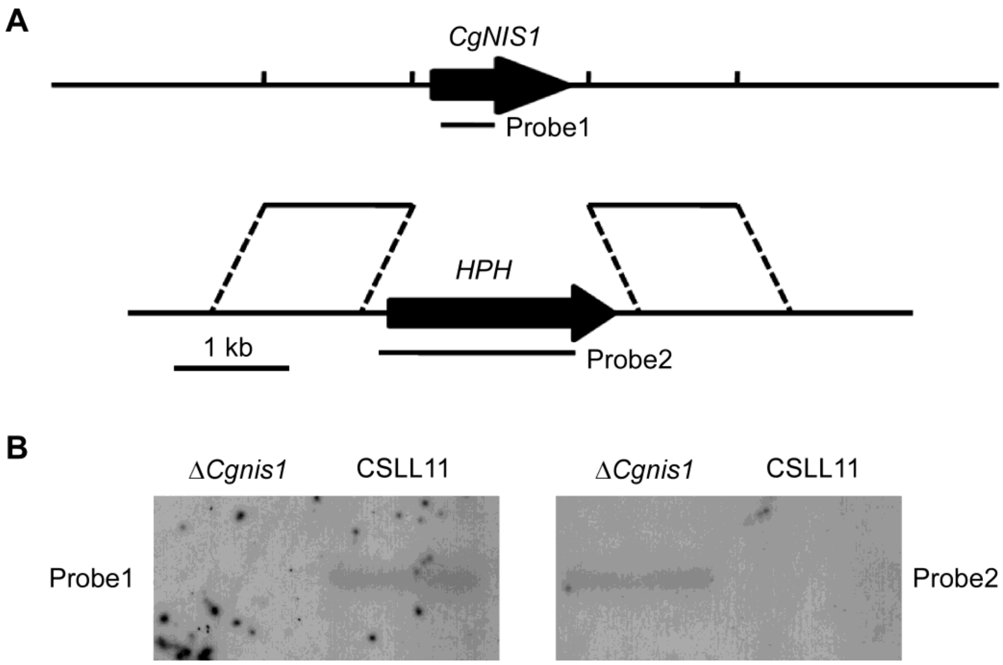


Figure S1. *CgNIS1* gene knockout

(A) A 492 bp fragment of the *CgNIS1* coding region were replaced by a 1.4 kb fragment containing the hygromycin B resistance cassette to create *CgNIS1* deletion mutant. (B) Southern hybridization analysis was used to validate the deletion of *CgNIS1* gene and the addition of a single copy integration of the *HPH* gene.

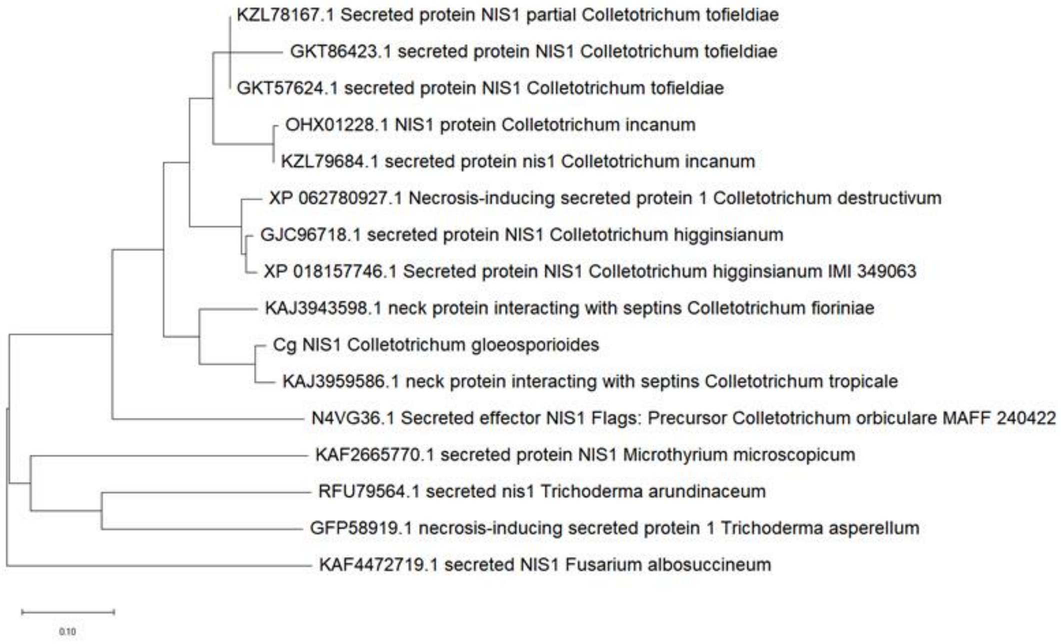


Figure S2. Phylogenetic analysis of NIS1 effectors in different fungi.

Table S1 Primers used in this study.

| Primer name | Primer Sequences (5'-3') | Remark |
|---|---|---|
| *NIS1*-UP-F+ | CTCGAGGCGCATCCTACCCGAATTTCTCAGCTT | *CgNIS1* deletion vector construction |
| *NIS1*-UP-R+ | GTCGACTTTGAATGATTTGTAGGGGGTTGGTGA | |
| *NIS1*-DOWN-F+ | ACTAGTATGCAGTACGACTTCATGAGCATCGGA | |
| *NIS1*-DOWN-R+ | GCGGCCGCTCGTTGTGTACTCCATGTCTTCTTTGC | |
| *NIS1*-F | ATGCAGTTCCGCGCTTCCATCGCCGCC | Amplification of *CgNIS1* probe for Southern blot |
| *NIS1*-R | TTACTGGCTGCCGACGTAGTTCTCGCT | |
| *NIS1*-C-F | GATAAGCTTGGAAGTAACGTTGAA TTTCGGAGAGCT | Complemented vector construction of *CgNIS1* |
| *NIS1*-C-R | AGAACTAGTTTACTGGCTGCCGACGTA GTTCTCGCT | |
| *NIS1*-GFP-F | GGTACCACCA TGCAGTTCCGCGCTTCCATCGCC | pBin::*CgNIS1*::eGFP vector construction |
| *NIS1*-GFP-R | CCCGGGAGATTACTGGCTGCCGACGTAGTTCTC | |
| *HPH*-F | GGAGGTCAACACATCAATG | Primer for *HPH* gene |
| *HPH* -R | CTCTATTCCTTTGCCCTCG | |

.
